# Design of an SVM Classifier Assisted Intelligent Receiver for Reliable Optical Camera Communication

**DOI:** 10.3390/s21134283

**Published:** 2021-06-23

**Authors:** Md. Habibur Rahman, Md. Shahjalal, Moh. Khalid Hasan, Md. Osman Ali, Yeong Min Jang

**Affiliations:** 1 Department of Electronics Engineering, Kookmin University, Seoul 02707, Korea; rahman.habibur@ieee.org (M.H.R.); mdshahjalal26@ieee.org (M.S.); osman@kookmin.ac.kr (M.O.A.); 2 Department of Electrical and Computer Engineering, Stevens Institute of Technology, Hoboken, NJ 07030, USA; khalidrahman45@ieee.org; 3 Department of Electrical and Electronic Engineering, Noakhali Science and Technology University, Noakhali 3814, Bangladesh

**Keywords:** support vector machine (SVM) classifier, intelligent receiver, optical camera communication (OCC), image sensor

## Abstract

Embedding optical camera communication (OCC) commercially as a favorable complement of radio-frequency technology has led to the desire for an intelligent receiver system that is eligible to communicate with an accurate light-emitting diode (LED) transmitter. To shed light on this issue, a novel scheme for detecting and recognizing data transmitting LEDs has been elucidated in this paper. Since the optically modulated signal is captured wirelessly by a camera that plays the role of the receiver for the OCC technology, the process to detect LED region and retrieval of exact information from the image sensor is required to be intelligent enough to achieve a low bit error rate (BER) and high data rate to ensure reliable optical communication within limited computational abilities of the most used commercial cameras such as those in smartphones, vehicles, and mobile robots. In the proposed scheme, we have designed an intelligent camera receiver system that is capable of separating accurate data transmitting LED regions removing other unwanted LED regions employing a support vector machine (SVM) classifier along with a convolutional neural network (CNN) in the camera receiver. CNN is used to detect every LED region from the image frame and then essential features are extracted to feed into an SVM classifier for further accurate classification. The receiver operating characteristic curve and other key performance parameters of the classifier have been analyzed broadly to evaluate the performance, justify the assistance of the SVM classifier in recognizing the accurate LED region, and decode data with low BER. To investigate communication performances, BER analysis, data rate, and inter-symbol interference have been elaborately demonstrated for the proposed intelligent receiver. In addition, BER against distance and BER against data rate have also been exhibited to validate the effectiveness of our proposed scheme comparing with only CNN and only SVM classifier based receivers individually. Experimental results have ensured the robustness and applicability of the proposed scheme both in the static and mobile scenarios.

## 1. Introduction

In recent years, optical camera communication (OCC) has been considered a potential wireless access option owing to the recent advancement in the luminous efficacy of light-emitting diode (LED) technology. At present, a dense formation of LED-based lighting infrastructures is being observed almost everywhere owing to offering low-power consumption, low-cost, and large optical spectrum immune to electromagnetic interference [1,2,3]. The large cost-free optical spectrum has created a new venture for resolving data congestion into the radio frequency (RF) spectrum. Thus, LEDs have become a significant option for illumination and communication simultaneously. In contrast, the successive improvement of complementary metal-oxide-semiconductor (CMOS) technology has integrated a new dimension in OCC development providing high pixel resolution and high-speed built-in camera inherited smart devices [4,5]. Therefore, OCC has become a promising indoor and outdoor communication technology.

Recently, with the enrichment of OCC technology, it has opened up a new frontier for the future massive connected network. Recently, academia and industry both have a surge of interest in taking advantage of OCC technology. OCC has substantial potential as most of the in-road vehicles are camera mounted and numerous LED infrastructures are installed in the outdoor environment. A variety of beneficial applications are expected to be achieved leveraging OCC technology in some other prospective areas such as robotics, automation, and e-Health. Furthermore, to deploy a 5G communication system effectively and to meet the requirement of the upcoming 6G communication system, OCC provides ample opportunity. In addition, OCC is suitable for the safety applications which require low BER and low latency [6,7,8,9,10]. While this technology is being continuously improved, it is not yet adequate to coronate OCC technology commercially in the initially envisioned areas. Currently, the artificial intelligence (AI) based approach has been adopted to enhance different performance features, for example, data rate, SNR, and communicating distance [11].

In the traditional scheme, the stream of data are converted into an optical signal being modulated using LEDs. Various modulation techniques have been proposed so far including both single-level intensity modulation schemes such as ON-OFF keying (OOK), under-sampled frequency shift ON-OFF keying (UFSOOK), under-sampled phase shift ON-OFF keying (UPSOOK), and spectral-efficient transmission scheme techniques such as color shift keying (CSK) and multi-level intensity modulation. Afterwards, the modulated signal is sent through a wireless medium [12,13,14,15,16]. The transmitted data are captured in the image sensor receiver. Deep learning techniques, notably convolutional neural network (CNN), are employed at the receiver side to detect LED, and the data bits are retrieved using several image processing techniques. Recently, it has been reported that stripe distortion can be effectively reduced when CNN is employed to extract features from the bright and dark striated frames during decoding [17,18]. The key issues that can characterize the OCC performance are broadly investigated in [19]. A new study have been conducted to improve decoding methods. They demonstrated differentiating multi-valued data more correctly for intelligent transport systems [20]. In addition, the authors have also demonstrated the variation of OCC performance based on the key performance parameters. Machine learning techniques at the receiver side have also been investigated due to the low computational complexity compared with the deep learning algorithms [21]. Authors in [22] used a support vector machine (SVM) in 8-superposed pulse amplitude modulation (8-SPAM) and direct-current-biased optical orthogonal frequency division multiplexing-based visible light communication (VLC) systems for LED detection purpose. SVM utilized optical barcode detection technique based on VLC have been studied in [23]. In [24], the authors have described SVM for constellation classification in two kinds of geometrically shaped 8-quadrature amplitude modulation for seamlessly integrated fiber and the VLC system. However, in all the traditional detection schemes, all the detected LEDs have been considered as data transmitting LEDs. In practice, many unwanted LED regions can be detected, which demolishes the robustness of the communication performance. For object detection purposes, CNN outperforms the other machine learning techniques. But, it cannot detect the actual transmitting LED regions deleting other unwanted LED regions from the camera frame because it does not consider spatial information of the object. Therefore, it fails to differentiate actual data transmitting LED from other unwanted LEDs [25]. Consequently, the data decoding process gets vulnerable in practical scenarios such as V2V and robotics in the presence of many LEDs in the field of view (FOV) of the camera. This seems to be a large roadblock towards the potential application of OCC technology.

**Figure 1:**
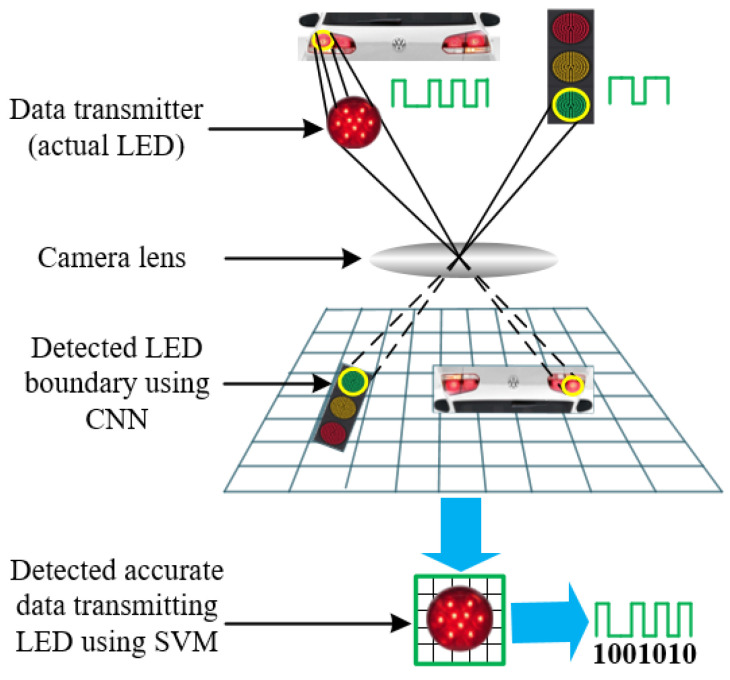
Scenario of the intelligent receiving system for OCC.

To the best of our knowledge, it is not investigated yet how a receiver can recognize actual data transmitting LED region from the frame among other unwanted LED regions and decode data intelligently from that accurate region in the OCC case. Therefore, in this paper, a novel intelligent camera receiver system employing SVM, a supervised learning model, along with CNN for OCC, has been designed. Our designed receiver system is capable of decoding data bits separating an accurate data transmitting LED region among various light-emitting sources present in the camera frame. Taking into account the visual flickering, we have modulated the optical beam by the FSOOK modulation technique, a low-cost and low-complexity modulation technique, which encodes bits into different frequencies [26]. This modulated optical signal was transmitted through a wireless medium to the receiver in the presence of other blinking LEDs. The scenario of how the proposed intelligent camera receiver works is depicted in Figure 1. Based on our scheme, we have developed a new data decoding algorithm for FSOOK based OCC. Our designed receiver first detects every LED region employing CNN. From each region, features are extracted to train the SVM classifier. Using the trained classifier model, the camera successfully separates accurate regions for data decoding purposes. As a result, unwanted LED regions are removed which will result in low computational complexity and mitigation of neighboring interference.

The remainder of this paper is structured as follows: Section 2 describes the SVM classifier. The overall OCC system including the modulation technique and algorithm for decoding data intelligently from the recognized region has been illustrated in Section 3. We have demonstrated how the data set has been prepared using extracted features in Section 4. In Section 5, we have elaborately discussed the experimental results to justify our scheme. Finally, we concluded our article in Section 6.

## 2. Support Vector Machine

**Figure 2:**
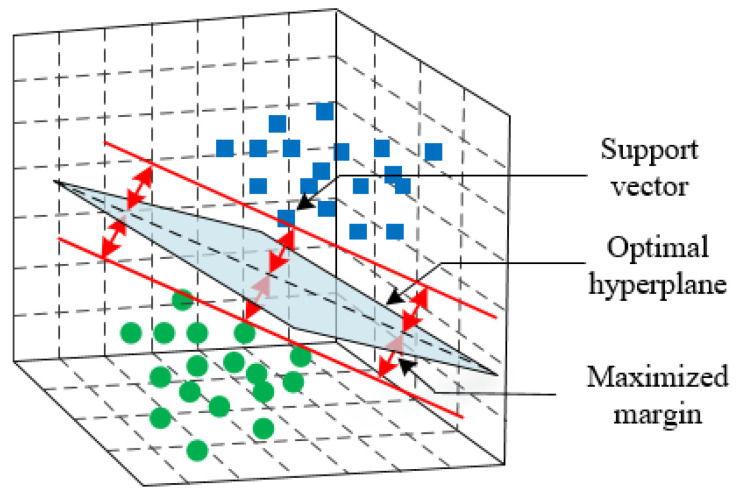
Illustration of SVM classifier.

The SVM has been considered one of the most widely used and accurate classification algorithms than others after being introduced in 1990 within the machine learning community [27]. Fundamentally, it is a supervised learning algorithm whose main objective is creating an optimal hyperplane between two classes of data set focusing on the training samples even if for a miniature training set. The hyperplane acts as a decision boundary to categorize the data in different classes. In Figure 2, points near the hyperplane, called support vectors, are used to determine the optimized hyperplane. For a given training sample $\left(x_{1},y_{1}\right),\left(x_{2},y_{2}\right),\ldots ,\left(x_{m},y_{m}\right)$ where class labels are represented by

(1) $$y_{i}=\left\{+1,-1\right\},\forall i\epsilon \left\{1,2,3,\ldots ,m\right\}.$$

The optimal separating hyperplane is mathematically formulated as [28]

(2) $$\theta^{T}x_{i}+b=0,$$

where $\theta =\left[\theta_{1},\theta_{2}\ldots ,\theta_{m}\right]$ is an *m*-dimensional vector of weights, and $x_{i}=\left[x_{0},x_{1},\ldots ,x_{m}\right]$ is an *m*-dimensional input vector, where $x_{0}=1$ by convention, and *b* is called the biasing unit. The optimization problem associated with finding the hyperplane can be expressed as follows:

(3) $$\frac{1}{2}{∥\theta ∥}^{2}=\frac{1}{2}\theta^{T}\theta ,$$

which is subjected to

(4) $$\theta^{T}x_{i}+b\geq +1\;\;\;\;\mathrm{for}\;\;\;\;y_{i}=+1.$$

(5) $$\theta^{T}x_{i}+b\leq +1\;\;\;\;\mathrm{for}\;\;\;\;y_{i}=-1.$$

The final nonlinear decision function can be obtained as follows:

(6) $$f\left(x\right)=sign\left(\sum_{i=1}^{m}\alpha_{i}\left(\theta^{T}x_{i}\right)+b\right).$$

To generalize the SVM classifier about complex data features, SVM uses a technique called Kernel $k(x_{i},x)$. The value $k(x_{i},x)$ corresponds to $\varphi \left(x_{i}\right).\varphi \left(x\right)$, which maps linearly non-separable patterns into a higher dimension feature space. The commonly used kernel functions, e.g., linear, polynomial, sigmoid, and radial basis function (RBF), are briefly described in Table 1. Finally, the decision function can be modified as follows:

(7) $$\begin{array}{cc} f(x) & =sign\left(\sum_{i=1}^{m}\alpha_{i}\left(\varphi \left(x_{i}\right)\cdot \varphi \left(x\right)\right)+b\right). \end{array}$$

## 3. OCC System Overview

### 3.1. OCC System Architecture

**Figure 3:**
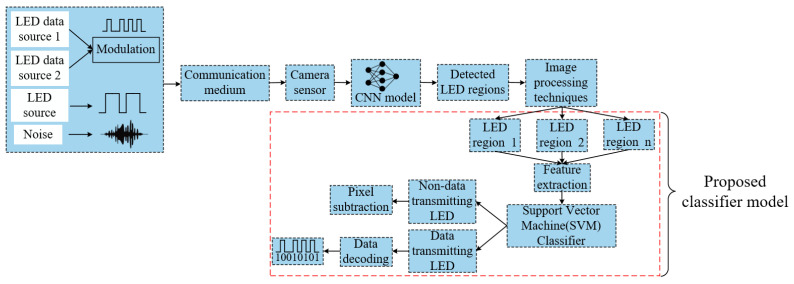
Overall OCC architecture with the proposed classifier model.

In Figure 3, the block diagram delineates the overall OCC architecture in addition to the proposed classifier model. The structure mainly consists of two parts: the transmitter and the receiver, but, unlike the traditional receiver system, the receiver part includes some additional features that assist with differentiating the accurate data transmitting LED over other LEDs. On the transmitter side, numerous LED regions were present. The LED optical signal was modulated by FSOOK modulation at a frequency level between 2–4 kHz before sending to the receiver. While the modulation is performed, the LED blinking should not be perceivable to the human eye. Since the perceivable flickering rate is up to 200 Hz [29], the modulation frequency used in FSOOK attenuates the flickering issue in a significant margin. With regard to the transmitting LEDs and other sources of interference, they are not modulated in that particular frequency. The projection of all these light-emitting sources was captured by the image sensor. Interference sources were dispelled when a trained CNN model is used. All the possible LED sources were detected and segmented using image processing techniques. Due to the rolling shutter effect of the CMOS based image sensor, each image frame gets striated of white and black shade. Afterwards, necessary geometrical features have been extracted to classify and recognize accurate data transmitting regions from the range of all possible regions.

### 3.2. FSOOK Modulation

**Figure 4:**
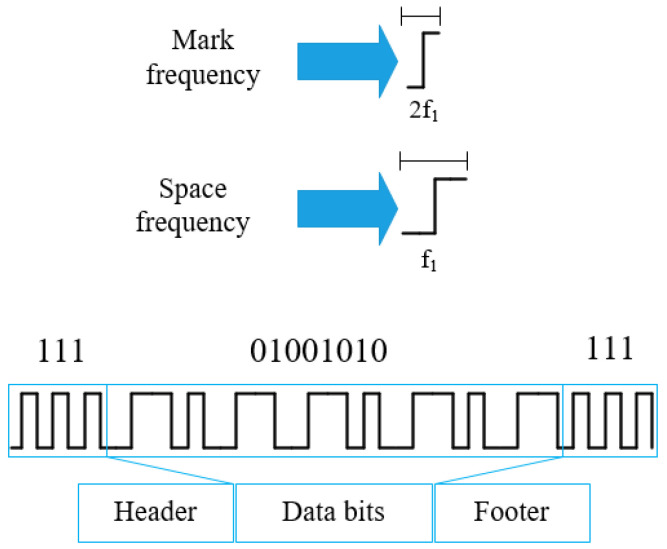
Illustration of the FSOOK modulation technique and data packet architecture.

It is a modulation scheme that encodes bit ‘1’ and bit ‘0’ with two different frequencies known as mark and space frequency, respectively [26]. The header and footer are appended at the beginning and ending of data bits respectively to identify the data packets. In order to create distinctions among header, footer, and data bits, we use the synchronization bit pattern ‘111’ as the header and footer. Due to the rolling shutter effect, we get a sequence of bit patterns in each data packet depicted in Figure 4.

### 3.3. Proposed SVM Classifier Assisted Intelligent Receiver

#### 3.3.1. LED Region Detection by a Convolutional Neural Network

**Figure 5:**
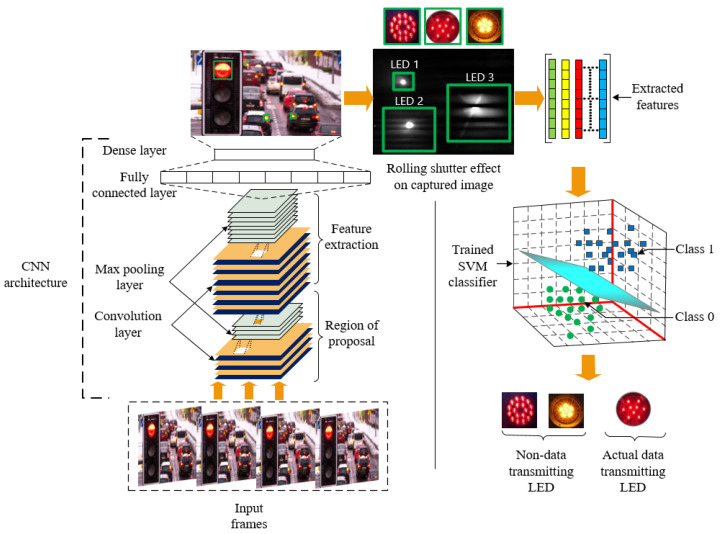
Workflow inside the proposed intelligent OCC receiver.

The workflow of the proposed intelligent receiver system is depicted in Figure 5. The receiver system consists of a CMOS based image sensor that collects optical signals transmitted through the optical channel. We have used the ’Microsoft LifeCam Cinema Webcam’ as the receiver during the experiment. Since the captured color image is formed by the combination of three color channels, we have denoted the capture image by $I_{u\times v\times 3}$, where *u* and *v* represent the height and width of the images. The captured image is resized into 512×512, and the resized image is given as an input to the object classifier designed by the CNN algorithm. These algorithms are eligible to extract image features automatically and can learn from those features effectively. To perform the LED detection, the CNN model has been trained by the images of our used LED captured in our lab environment. We have taken 2500 images to build our data set. Images from different angles and distances are considered in our data set to train the CNN model more accurately. The CNN model recognizes all light source regions $I_{u1\times v1\times 3}^{1},I_{u2\times v2\times 3}^{2},I_{u3\times v3\times 3}^{3},\ldots ,I_{un\times vn\times 3}^{n}$, where *n* is the total number of LEDs in the frame by performing the functions like region proposal, feature extraction, classification, etc. Several layers inside CNN, namely convolutional layers, pooling layers, and fully connected (FC) layers, perform those functions, but the convolutional layer is the main part of CNN. The convolutional layers are composed of a convolutional kernel that is used to generate the proposed region and feature maps from the original image. The convolutional kernel can be of many sizes. In our training phase, we have used ten 4×4 convolutional filters in the first convolutional layer and twenty 4×4 convolutional filters in the second convolutional layer. The convoluted output is estimated by the following equation [30]:

(8) $$y=\sum_{q=0}^{q-1}\sum_{p=0}^{p-1}\left(w_{pq}x_{a+1,b+p}+\Theta \right),\left(0\leq a\leq a_{f},0\leq b\leq b_{f}\right).$$

Max pooling with a 2×2 filter and 2 strides is further used in another two layers to reduce the spatial size of the warped images and computation in the network resulting in being less prone to overfitting preserving all features. This pooled feature map was flattened into one column while passing through the FC layer. Here, the FC layer tries to determine the most correlated features of a particular class. The non linear softmax function is used to classify LEDs at the dense layer from other objects in the image frame. The ’Adam’ optimizer has been used to optimize our CNN model. As part of the optimization process, the cost function, conventionally called loss function, is required to calculate the error at each cycle of the training of our CNN model. Among various cost functions, we have considered ’binary cross-entropy’ function in our work since we needed to recognize whether the object in the image frame is the LED or not. Now, using the chosen optimizer and cost function, the CNN model tries to reduce the error by updating weights in each layer of the neural network.

#### 3.3.2. Accurate Data Transmitting LED Region Separation

In Figure 5, the detected LED regions with bounding boxes are the output of the CNN. The bounding box LED objects are then processed employing several image processing techniques. At first, each LED image is converted into a grayscale format $f_{p\times q}$ to specify the distinct pixel intensity values of the stripes generated in the image sensor. Next, a 2×2 kernel full of ones is used to reduce the shape of the projected stripes by removing small anomalies near the stripe boundaries. To make the receiver an intelligent and robust retrieval system, the system needs to respond by differentiating accurate LEDs as quickly as possible. Therefore, the selection of features is very important. The extraction of appropriate features is very challenging since the objects are almost the same. LED objects of the same type can have a different shape, stripe pattern based on the communication distance, camera frame rate, and mark and space frequency. To analyze the different geometrical shapes, computation of the features from the contour line has been considered significant distinguishable features [31]. Contours are the outline that is designed using the edges of the object to represent the shape. They contain some geometrical attributes that are effective to recognize and segment objects. After drawing a contour on each separated region, a feature set $\left[M_{00},S_{T},P_{R},N_{L}\right]$ is formed that includes zero order moments or area of LED region, no. of stripes per LED region, perimeter of combined stripes contour, and no. of line segment of combined stripes contour, respectively. Based on the extracted input features, a trained SVM classifier detects accurate data transmitting LED regions.

#### 3.3.3. Data Decoding Method from the Accurate LED Region

After recognizing accurate data transmitting LED regions, the data bits have been decoded from the accurate LED regions by analyzing the produced bright stripes and dark stripes on the image frame. The overall demodulation scheme of our proposed intelligent receiver is shown in Algorithm 1. In Algorithm 1, the detection of LED regions using CNN to the separation of accurate data transmitting LED region have been pointed out from step 1 to step 9. From each separated region, normalized intensity value $Z_{i}$ is measured where *i* specifies the *i* th row of total *p* no. of rows in the separated accurate LED region. Now, the median value of the normalized intensities is calculated and set as threshold $Z_{th}$. Now, we have assigned $Z_{i}$ = ‘1’ if $Z_{i}>Z_{th}$ and vice versa to represent the ‘ON’ state, i.e., bright stripe and the ‘OFF’ state, i.e., dark stripe, respectively. In our experiment, the frequency to encode bit ’1’ is chosen as double that of the frequency to encode bit ‘0’. Therefore, the time period for the encoding bit ‘1’ is half that of encoding bit ’0’ as well. This leads to the variation in the no. of pixel rows in the image frame, where bright stripes and dark stripes are generated, concerning bit ‘0’ and bit ‘1’. Thus, we have calculated the no. of rows, which is represented as width, of successive ‘OFF’ and ‘ON’ states sequentially using ‘OpenCV’ module in Python, and they are denoted as $W_{0}$ and $W_{1}$. Finally, if both the width is greater than the ratio (δ) of each row read out time and half of the time period to encode bit ‘1’, the output is derived as bit ‘0’; otherwise, it is derived as bit ‘1’.

**Algorithm 1** Demodulation Scheme at the Receiver.**Input:** Captured image of all LEDs state.**Output:**$x_{o}=$ “11101011...” from accurate data transmitting LED region.1: read each RGB image $I_{u\times v\times 3}$;2: detection of each LED region by CNN and separate regions $I_{u1\times v1\times 3}^{1},I_{u2\times v2\times 3}^{2},I_{u3\times v3\times 3}^{3},\ldots ,I_{un\times vn\times 3}^{n}$;3: convert to grayscale image $I_{u\times v\times 3}\to f_{p\times q}\left(gray\right)$;4: draw contour on each of the $f_{p*q}^{n}$;5: **for**
i=0: no. of separated LED region **do**
6:     extract features $\left[M_{00},S_{T},P_{R},N_{L}\right]$;7:     pass features to trained SVM classifier;8:     recognize accurate data transmitting LED region;9: **end for**10: normalize each row intensities’ values $\sum_{i=1}^{p}Z_{i}$;11: set median of normalized values as threshold $Z_{th}$;12: **for**
i=0:p
**do**13:     **if** $Z_{i}>Z_{th}$ **then**14:         assign $Z_{i}=$ “1”;15:     **else**16:         assign $Z_{i}=$ “0”;17:     **end if**18:     calculate width $W_{0}$ of successive $Z_{i}=$ “0” and width $W_{1}$ of successive $Z_{i}=$ “0”;19: **end for**20: **if**
$W_{0}>\delta \;and\;W_{1}>\delta$
**then**21:     set $x_{o}=$ “0”;22: **else**23:     set $x_{o}=$ “1”;
24: **end if**

## 4. Data Set Preparation

The prime objective of our proposal is to enable the camera to intelligently determine data transmitting LED and communicate with this LED. Hence, the camera needs a trained AI-based classifier by which it can perform the required task. To train the classifier, a data set is needed which contains some necessary features from the bounding boxes of detected LED regions. In order to build the data set, 1000 preprocessed bounding box images of the LED region are used. The experimental parameters that are used to extract the features are listed in Table 2. Input feature vectors are constructed considering different situations such as varying distance from LED to the camera and moving LED from left to right. However, before performing feature extraction, a closing operation is applied on the selected regions to combine all the neighboring stripes, and the contour is drawn on the shape by combined stripes. It is a morphological filtering technique that is composed of gray level dilation followed by gray level erosion taking the input of the selected regions and a structuring element. The closing operation can be expressed as [32]

(9) $$f_{p\times q}^{n}\cdot b_{m\times n}=\left(f_{p\times q}^{n}⊕b_{m\times n}\right)\cdot b_{m\times n},$$

where $f_{p\times q}^{n}$ is nth region, and $b_{m\times n}$ is the structuring element of which m,n≪a,b.

**Figure 6:**
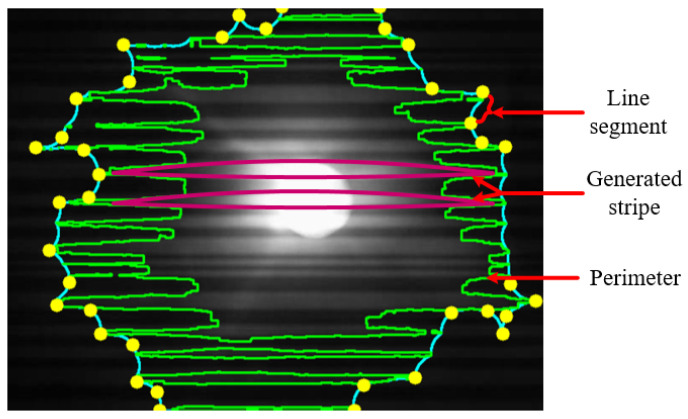
Example of a single LED image frame where all the generated stripes are combined together and a contour is formed.

In Figure 6, we have presented a single frame where the shape of combined stripes contour is shown. For each projected LED, either data transmitter or unwanted LED, we found this type of shape of the contour formed based on the combined stripe. Analyzing the geometrical pattern of the contour, there is considerable change in the pattern when the communication distance is varied or mark and space frequency have been changed. Hence, based on the contour pattern of the data transmitting LED region and other unwanted LED regions, we have selected several features that are elaborately described in the next section.

### 4.1. Zero Order Moments or Area of LED Region ($M_{00}$)

The moment is a unique feature that defines the relationship between pixels of the corresponding objects with their surroundings. For many years, this feature is being used to recognize objects from image frames. Fundamentally, it computes the weighted average intensity of the pixels, and, therefore, it cannot be applied on a single pixel except a definite area of the projected object [33]. For an image, $f_{p\times q}$ the moment of order (x,y) can be defined as

(10) $$M_{xy}=\sum_{p=0}^{m}\sum_{q=0}^{n}\sigma x^{p}y^{q}f_{p\times q};x,y=0,1,2,\ldots ,$$

where $\sigma x^{p}y^{q}$ denotes moment weighting kernel. Different image properties lie in the raw moments. In our case, we have denoted moment of order (0,0) as an area of the combined stripes for data transmitting LED.

### 4.2. No. of Stripes per LED Region ($S_{t}$)

Since LED modulated data results in dark and bright stripes in the captured image, the number of stripes and the width plays an important role in recovering the data stream from the image frame. The number of stripes is varied proportionally with the size of LED, mark, and space frequency of LED, and also the distance between the camera and LED [16]. It is noticed that high-speed cameras with high read-out capability outperform in terms of communication speed. The width of the bright and dark stripes can be theoretically determined as [19]:

(11) $$S_{T}=\frac{1}{ft_{r}},$$

where *f* represents mark or space frequency, and $t_{r}$ is the time needed to read-out a single pixel of the image.

### 4.3. Perimeter of Combined Stripes Contour ($P_{r}$)

A contour is drawn considering all the stripes generated by the LED blinking and rolling shutter effect of the camera. Hence, the arc length of the enclosed contour varies according to the number of stripes. Thus, the perimeter is also considered a significant feature for our data set.

### 4.4. No. of Line Segment of Combined Stripes Contour ($N_{L}$)

In 1973, the Douglas–Peucker algorithm developed by D. Douglas and T. Peucker splits a curve into line segments depending upon the specified precision (ϵ). It determines the dominant point on the curve by digitizing the original curve and generates a similar curve of fewer points [34]. We have used this algorithm to relate the number of line segments with the no. of stripes. It is noticeable that, with the variation of LED on-off frequency and distance, getting accurate contour of the object is difficult. Thus, errors may be present in determining the number of stripes. Combining all stripes, we segmented the combined contour using the Douglas–Peucker algorithm based on the corner point of each stripe.

**Figure 7:**
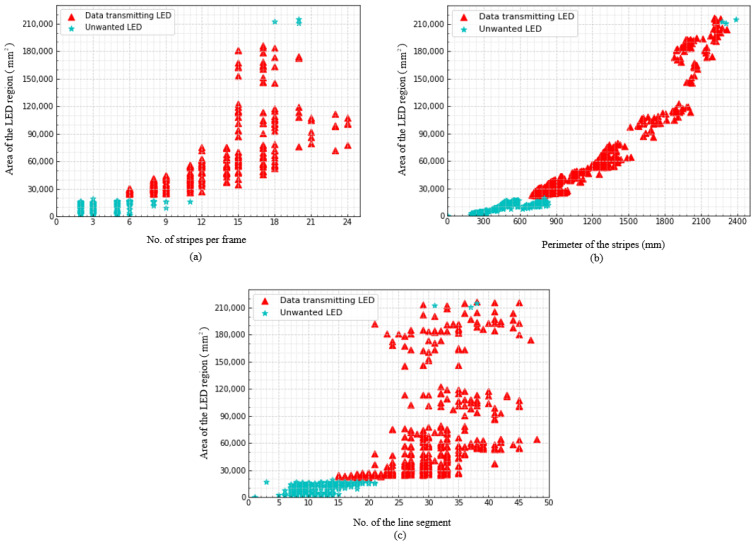
Scatter plot of selected features (**a**) area vs. no. of stripes per LED region; (**b**) area vs. perimeter of the combined stripes contour; and (**c**) area vs. no. of the line segment of the combined stripes contour.

The scatter plot of the selected features is shown in Figure 7. In the figures, the distribution of features shows a clear distinction among data transmitting LED and unwanted LED which affirms our motive to integrate an SVM classifier in the conventional receiver system.

## 5. Experimental Results

In this section, the performance of our proposed scheme for OCC receivers has been demonstrated explicitly. The overall performance has been measured experimentally, and the experiments were conducted considering different conditions. We have taken a maximum of three LEDs as data transmitting LEDs for our experiment. The distance among transmitters was set to 5 cm when multiple transmitters were used during the experiment. The whole decoding procedure of the receiver side has been performed and analyzed in the Python 3.7 environment. Firstly, we have examined the classifier performance to classify and recognize the accurate data transmitting LEDs by varying kernel functions of the SVM classifier. During the experiment, we have used three LEDs among which the no. of actual data transmitting LEDs have been varied. We have also varied the communication distance (10 cm to 60 cm) to justify the effectiveness of our scheme. However, to examine the efficiency of separating accurate LED regions intelligently, the SVM classifier performance has been investigated demonstrating the receiver operating characteristic (ROC) curve and other key performance parameters, such as precision,recall,andF1score. These parameters are defined as

(12) $$\begin{array}{cc} \; & Precision=\frac{True\;positive}{True\;positive+False\;positive}=\frac{True\;positive}{Total\;predicted\;positive},\\ \; & Recall=\frac{True\;positive}{True\;positive+False\;negetive}=\frac{True\;positive}{Total\;actual\;positive},\\ \; & F1\;score=2\times \frac{Recall\times Precision}{Recall+Precision}. \end{array}$$

We have also evaluated the performances from the communication perspective. It shows satisfactory performance on the BER, data rate, and inter-symbol interference (ISI).

### 5.1. Classifier Performance

**Figure 8:**
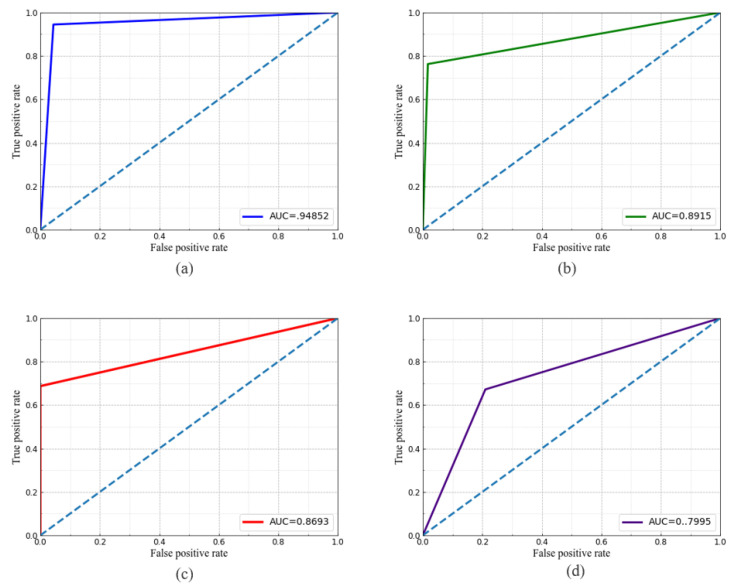
ROC curve of SVM classifier for (**a**) RBF kernel; (**b**) linear kernel; (**c**) polynomial kernel; and (**d**) sigmoid kernel.

We have classified accurate data transmitting region from all the possible captured LED regions detected by CNN using the trained SVM classifier. Based on the selected features, the SVM classifier provides an accurate LED region for demodulation. Thus, the other communication performances are greatly dependent on the classification that results in separated LED regions. Therefore, to evaluate classifier performance, we have shown the ROC curve for different kernel functions as shown in Figure 8. The communication distance and the exposure time $T_{e}$ were set to 30 cm and 976.6 μs, respectively. In addition, two LEDs have been used where one was data transmitting LED. The area measured under the ROC curve known as AUC is larger for the RBF kernel than others. Consequently, the classification accuracy is also larger for the RBF kernel.

**Figure 9:**
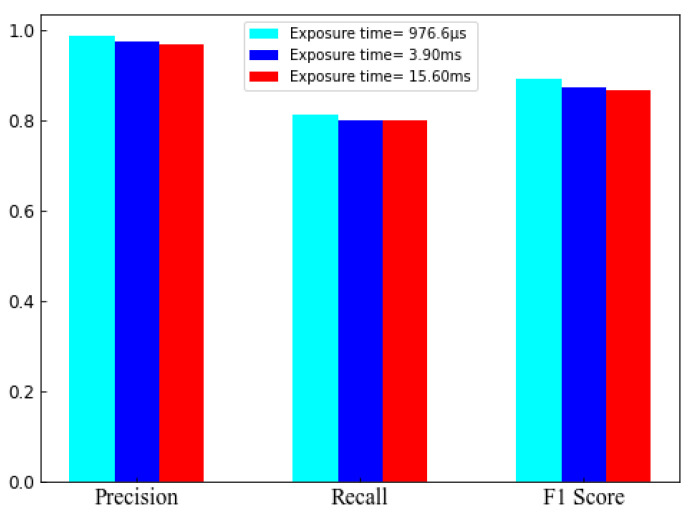
Comparison among precision, recall, F1 score varying exposure time ($T_{e}$).

In Table 3, we have presented overall accuracy and measured AUC values considering the different kernels and no. of data transmitting LEDs. To evaluate classifier performance, one additional non-data transmitting LED has been used with the data transmitting LEDs for each case. During the experiment, for a certain number of image frames, we have calculated the amount of ’TRUE’ data transmitting LED and amount of ’FALSE’ data transmitting LED recognized by the classifier. We have also calculated the amount of ’TRUE’ non-data transmitting LED and amount of ’FALSE’ non-data transmitting LED. Later, we have calculated accuracy and AUC using programming in the Python 3.7 environment. The maximum accuracy is recorded as 94.92% for the RBF kernel and the lowest accuracy of 79.87% is achieved for the sigmoid kernel. Moreover, we have also given a bar plot to show the comparison among key performance parameters varying exposure time $T_{e}$, while the amount of data transmitting LED was one, as shown in Figure 9. For the exposure time 976.6 μs, all the performance indexes are high. However, the achieved performance has validated that our scheme is capable of recognizing accurate LED regions.

### 5.2. BER, Data Rate, and ISI Analysis

**Figure 10:**
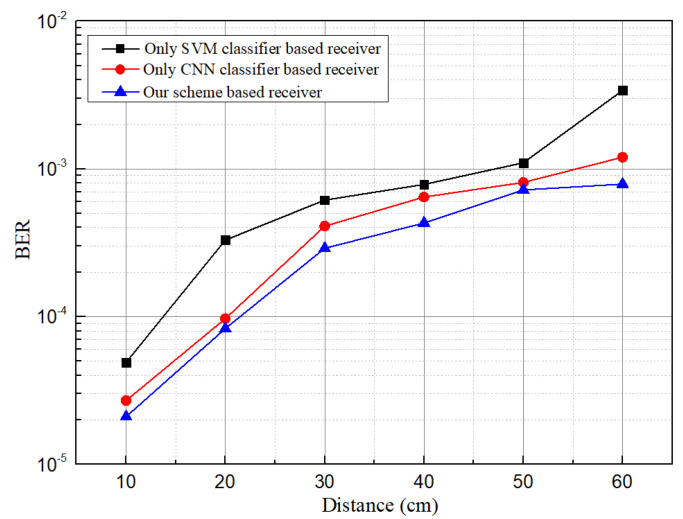
Comparison of bit error rate (BER) among receivers using only SVM, only CNN, and our proposed scheme.

After classifying accurate data transmitting LED, data bits have been decoded from the cropped region using a decoding algorithm described in Algorithm 1. We have decoded data and evaluated the communication performance of our proposed intelligent receiver. According to our scheme, the receiver intelligently selects accurate LED regions so the probability of interference from other sources or reflection from the surrounding surface have been reduced. Figure 10 depicts the measured BER performance against communication distance for the receiver employing only an SVM classifier, only CNN, and our proposed scheme. A significant reduction in BER has been observed when the SVM classifier along with CNN is used. At the maximum distance of 60 cm, the BER increases up to 7.9×$10^{-4}$, which is below the forward error correction (FEC) limit of $3.8\times 10^{-3}$. At this point, only the SVM based receiver shows BER above the FEC limit, whereas the CNN based receiver is very close to the FEC limit. It signifies the effectiveness of our proposed scheme.

**Figure 11:**
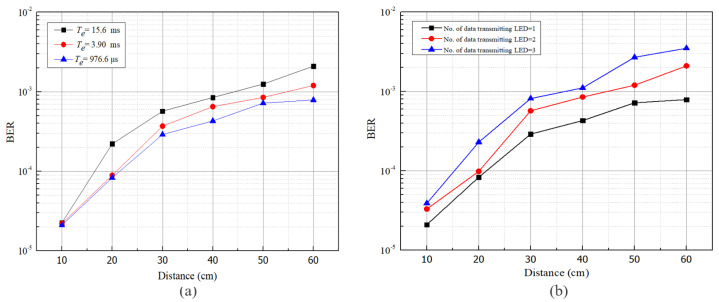
BER analysis by varying (**a**) exposure time ($T_{e}$) and (**b**) no. of data transmitting LEDs.

The BER performance varies based on exposure time. The effect of changing exposure time $T_{e}$ on BER for a single data transmitting LED keeping two unwanted LED regions has also appeared in Figure 11a. With the increase of exposure time, BER also increases due to the interfering lights from other sources. Although BER has increased with the increase of the exposure time $T_{e}$, the maximum BER at the maximum distance is below the FEC limit in our scheme. We have also investigated BER performance by varying the number of LEDs, as shown in Figure 11b. When the amount of actual data transmitting LED increases, efficient separation of LED regions has an enormous influence on BER. Therefore, a significant change in BER has been found when the amount of data transmitting LED has been increased from one to three. When the amount of data transmitting LED is three, the BER has been achieved around $3.5\times 10^{-3}$ for $T_{e}=976.6$ μs.

**Figure 12:**
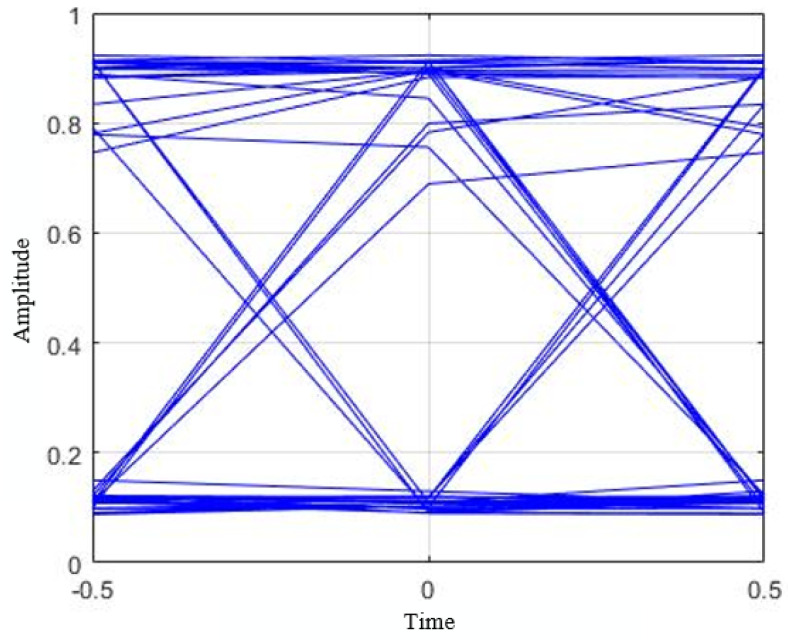
Eye diagram of SVM assisted intelligent OCC receiver while exposure time ($T_{e}$) is 977.6 μs.

Furthermore, the eye diagram depicted in Figure 12 at $T_{e}=976.6$ μs has ensured the low ISI at the receiver side. During multiple data transmitting LED region cases, if the receiver fails to differentiate distinct regions accurately, recovering a distinct pulse for a distinct bit can be difficult for the receiver. The large eye opening affirms the low ISI presence at separated regions classified by the SVM classifier.

**Figure 13:**
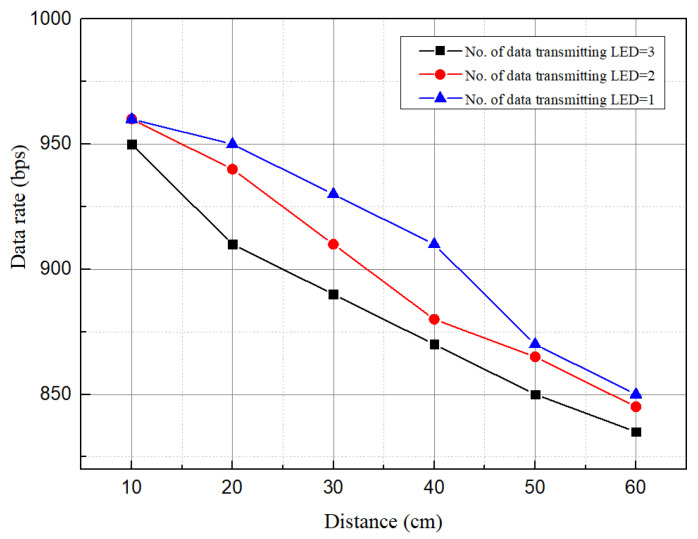
Data rate comparison varying no. of LEDs while $T_{e}$ is 977.6 μs.

Since the SVM classifier separates LED regions and data are decoded from the separated regions, it has a significant effect on data rate too. When communication distance is changed, some low intensity stripes may cancel out from the detected regions. A distinctive change in data rate is caused due to the change in no. of stripes in the LED regions. With the change in no. of LEDs, data rate variation against communication distance is shown in Figure 13. For a single LED, the maximum estimated data rate is 960 bps at distance 10 cm, and 830 bps is the lowest data rate, which is achieved at 60 cm for the three data transmitting LEDs. The data rate can be augmented significantly using an LED array with a BER lower than the FEC limit.

**Figure 14:**
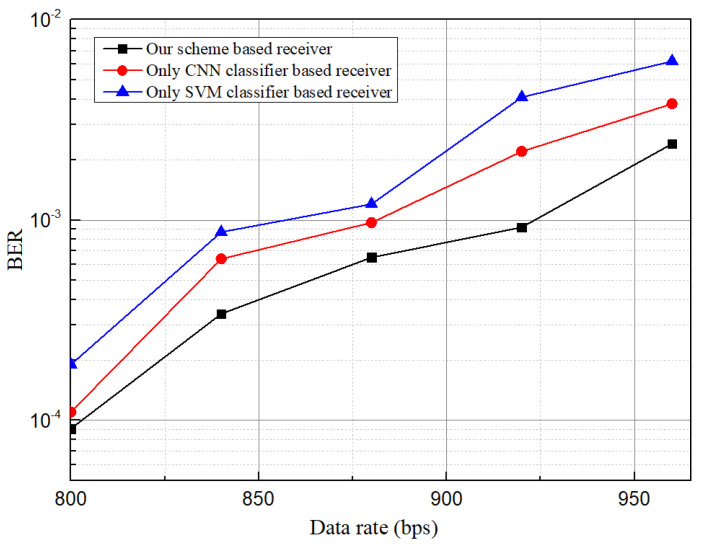
BER against data rate comparison among our scheme based classifier, receiver using only CNN classifier, and receiver using only SVM classifier.

However, some stripes can be recovered further by updating in the image processing steps at a fixed distance. It causes a change in the no. of erroneous and accurately decoded bits. Therefore, a significant change in the data rate and BER have been observed. Figure 14 illustrates BER vs. data rate performance while communication distance was 50 cm for three different cases. At each case, two LEDs were in data transmitting mode, and one was used as an unwanted region in the image frame. For achieving 960 bps, BER recorded for SVM-based receiver is beyond the FEC limit, while the CNN-based receiver shows more closeness to the FEC limit. Our scheme-based receiver can decode data at 960 bps at a BER of $3.5\times 10^{-3}$.

## 6. Conclusions

In the conventional OCC system, only CNN has been used to detect LED regions irrespective of any unwanted LED regions or multiple LED regions from multiple users. In this article, we have designed a scheme by which the camera receiver can differentiate accurate data transmitting LED among unwanted LED leveraging SVM classifiers additionally for the first time. The FSOOK modulation scheme has been used to modulate data that is sent by blinking of the LED accordingly. We have evaluated the overall performance elaborately demonstrating the ROC, and other key performance parameters of the classifier, as well as other communication performance features, such as BER, data rate, and ISI. The ROC of the SVM classifier affirms its efficacy to accurately classify LED. The BER analysis exhibits 10 times lower BER at the maximum communication distance (60 cm). In addition, by the eye diagram, we have also investigated ISI of the decoded data from the recognized LED region. Moreover, the data rate against a distance varying no. of LEDs has been observed for our proposed scheme. In addition, BER against the data rate is also discussed to ensure the necessity of our proposed scheme. However, it is perceptible from the overall analysis that our scheme is a novel approach for reliable OCC communication. Based on our scheme, a new frontier is expected to be outstretched for the OCC to deploy in future communication technology.

## Figures and Tables

**Table 1:** Kernel functions for the proposed system.

Type	Function
Linear	$x_{T}x_{i}+c$
Radial basis function (RBF)	$exp\left(\left|-\frac{{∥x-x_{i}∥}^{2}}{2\sigma^{2}}\right|\right)$
Polynomial	${\left(x_{T}x_{i}+c\right)}^{p}$
Sigmoid	$\tanh({\left(x_{T}x_{i}+c\right)}^{p})$

**Table 2:** Parameters used in the implementation of the proposed model.

Parameter	Value
LED diameter	10 mm
Camera exposure time	15.6 ms, 3.90 ms, and 976.6 μs
Camera frame rate	30 fps
Camera image resolution	1280×720
Mark and space frequency	4000 kHz and 2000 kHz
Learning rate	0.001
No. of epoch	30
Kernels	RBF, linear, polynomial, and sigmoid

**Table 3:** Classifier performance (accuracy and AUC) analysis with a different no. of LEDs.

No. of Data Transmitting LED		Linear	RBF	Polynomial	Sigmoid
One	Accuracy (%)	92.09	94.92	89.45	80.93
	AUC	0.89	0.94	0.87	0.79
Two	Accuracy (%)	90.05	93.10	87.91	81.36
	AUC	0.85	0.91	0.83	0.81
Three	Accuracy (%)	87.76	89.91	88.13	79.87
	AUC	0.84	0.88	0.85	0.77

## Data Availability

We have build our own data set in this study. Since further research are on processing, we cannot publish data set right now.
